# Supplementary material for: Negotiating acceptable termination of pregnancy for non-lethal fetal anomaly: a qualitative study of professional perspectives
Source: BMJ Open. 2018 Mar 1;8(3):e020815. doi: 10.1136/bmjopen-2017-020815 (PMC5855171; doi:10.1136/bmjopen-2017-020815)
Supplement: Author's manuscript [file bmjopen-2017-020815.draft_revisions.pdf]

BMJ Open is committed to open peer review. As part of this commitment we make the peer review history of every article we publish publicly available.

When an article is published we post the peer reviewers' comments and the authors' responses online. We also post the versions of the paper that were used during peer review. These are the versions that the peer review comments apply to.

The versions of the paper that follow are the versions that were submitted during the peer review process. They are not the versions of record or the final published versions. They should not be cited or distributed as the published version of this manuscript.

BMJ Open is an open access journal and the full, final, typeset and author-corrected version of record of the manuscript is available on our site with no access controls, subscription charges or pay-per-view fees (http://bmjopen.bmj.com).

If you have any questions on BMJ Open's open peer review process please email info.bmjopen@bmj.com

BMJ Open

Negotiating acceptable termination of pregnancy for non-lethal fetal anomaly: Professional perspectives. A qualitative study.

Journal:	*BMJ Open*
Manuscript ID	bmjopen-2017-020815
Article Type:	Research
Date Submitted by the Author:	29-Nov-2017
Complete List of Authors:	Crowe, Lisa; Newcastle University , Institute of Health and Society<br>Graham, Ruth; Newcastle University, School of Geography, Sociology and Politics<br>Robson, Stephen; Newcastle University, Institute of Cellular Medicine<br>Rankin, Judith; Newcastle University, Institute of Health & Society
**Primary Subject Heading**:	Reproductive medicine
Secondary Subject Heading:	Qualitative research
Keywords:	Fetal medicine < OBSTETRICS, Prenatal diagnosis < OBSTETRICS, Maternal medicine < OBSTETRICS, QUALITATIVE RESEARCH

SCHOLARONE™
Manuscripts

For peer review only

Negotiating acceptable termination of pregnancy for non-lethal fetal anomaly: Professional perspectives. A qualitative study.

Lisa Crowe,[1] Ruth H Graham,[2] Stephen C Robson,[3] Judith Rankin.[1]

[1]Institute of Health & Society, Baddiley-Clark Building, Newcastle University, NE2 4AX.

[2] School of Geography, Politics and Sociology, 5th Floor Claremont Bridge Building, Newcastle University, NE1 7RU.

[3] Institute of Cellular Medicine, 3rd Floor William Leech Building, Newcastle University, NE2 4HH

Corresponding Author: Lisa Crowe. Lisa.Crowe@ncl.ac.uk

Word count (main body): 3,999.

Abstract

For peer review only

Objective: This study aimed to explore, describe and understand medical and social care professionals' perspectives on the meanings and implications of non-lethal fetal anomaly and to evaluate the relationship between these meanings and participants' perceptions of termination of pregnancy for fetal anomaly (TOPFA).

Methods: Semi-structured interviews were undertaken with medical professionals (14 consultants in fetal medicine, obstetrics, neonatology and paediatrics) and social care professionals (nine individuals with roles supporting people living with impairment) from the North East of England. Analysis adopted an inductive thematic approach facilitated by NVivo.

Results: The overarching theme to emerge from the interview data was of professionals, medical and social care, wanting to present an acceptable self-image of their views on TOPFA. Professionals' values on 'fixing', pain and 'normality' influenced what aspects of moral acceptability they gave priority to in terms of their standpoint and, in turn, their conceptualisations of acceptable TOPFA. Thus, if a termination could be defended morally, including negotiation of several key issues (including 'fixing', perceptions of pain and normality), then participants conceptualised TOPFA as an acceptable pregnancy outcome.

For peer review only - http://bmjopen.bmj.com/site/about/guidelines.xhtml

Conclusion: Despite different professional experiences, these professional groups were able to negotiate their way through difficult terrain to conceptualise TOPFA as a morally acceptable principle. While professionals have different moral thresholds, no one argued for a restriction of the current legislation.

Keywords: UK; North East England; Termination of pregnancy; fetal anomaly; professionals; qualitative.

Strengths and Limitations:

- This qualitative study provides in-depth data on views on TOPFA from a previously unexplored professional group, social care professionals.
- The qualitative nature of this study allowed for the exploration of a sensitive research topic.
- The use of case study examples provided tangible examples with which to explore key issues in the process of negotiating moral acceptability of TOPFA.
- This study was conducted in the North East of England so generalisation cannot be assumed.

For peer review only

This work was supported by a Medical Research Council/ Economic and Social Research Council Integrated PhD studentship, grant number G0800128-3/1.

Introduction

Termination of pregnancy for fetal anomaly (TOPFA) is legal under the Abortion Act 1967, amended by the Human Fertilisation and Embryology Act (HFEA) (1) with no upper gestational limit if there “is a substantial risk that if the child were born it would suffer from such physical or mental abnormalities as to be seriously handicapped” (Clause E). In 2016, 3,208 terminations were carried out under Clause E, 2% of the total number.(2) The detection of fetal anomalies is likely to continue to increase due to improvements in fetal imaging and increasing risk factors for fetal anomalies (for example, obesity).(3-6) Complex issues emerge as medical professionals juggle multiple moral implications; judging the anomaly in question, offering choice, protecting themselves from prosecution while also providing care to parents.(7) These issues are coupled with the limited treatments in-utero, restricting the options for many parents to essentially TOPFA, or continuing an affected pregnancy.(8) Thus, medical professionals working in prenatal diagnosis have the potential to influence decision making processes being made about an affected pregnancy; understanding

For peer review only - http://bmjopen.bmj.com/site/about/guidelines.xhtml

professionals' views of TOPFA is therefore of crucial importance. This is especially significant given recent research that has found that care received by parents undergoing TOPFA was felt to not adequately meet their needs; for example, being caught between antenatal and postnatal care settings, yet belonging to neither.(9)

Medical professionals providing TOPFA deal with complex information when deciding whether to offer TOPFA or not.(10, 11) However, their knowledge and experience of living with disability and impairment tends to be more limited. This is argued to be of concern if assumptions about experience of impairment lead to disability being automatically equated to 'unhealthy'.(12) Similarly, those in the social care sector, involved in the support and care of those living with impairment, have more knowledge about experiences of impairment, but less insight into the decision making process that leads to TOPFA. Understanding the views of both medical and social care professionals about TOPFA is important not only to facilitate the provision of appropriate care and to provide support to those making reproductive decisions, but also to gain enhanced insight into how life with impairment is conceptualised from varied perspectives. To our knowledge, there is no research on the opinions of social care professionals on TOPFA.

Personal views and experiences affect professional behaviour and the views of professionals impact on the pregnant woman. For example, research suggests that

For peer review only - http://bmjopen.bmj.com/site/about/guidelines.xhtml

some women have felt they were counselled 'towards' TOPFA (13, 14), and other research has identified variations in counselling techniques.(15, 16) Religious affiliation has also been found to impact counselling practices.(17) If personal views impact on counselling practices, this may, in turn, influence decisions about TOPFA. Social care professionals have a limited voice in debates on TOPFA. Their views offer a different professional insight into what living with an impairment is like.

This study aimed to explore, describe and understand medical and social care professionals' perspectives on the meanings and implications of non-lethal impairment and to evaluate the relationship between these meanings and participants' perceptions of TOPFA.

Methods

This paper reports data collected as part of a larger study examining professionals' views on TOPFA.(18) The study utilised semi-structured interviews to collect data exploring the meanings of specific and complex issues relating to TOPFA. Four fetal anomalies were selected as case studies; isolated cleft lip, hypoplastic left heart (HLH), spina bifida and Down syndrome. These examples ensured that discussions included reference to a range of conditions, affecting physical and intellectual capacity and with

For peer review only - http://bmjopen.bmj.com/site/about/guidelines.xhtml

impact ranging from functionally minor to lethal. Participants were provided with an invitation letter, information sheet and consent form.

Two professional groups were recruited to the study. The first group was medical consultants working in the fields of obstetrics, fetal medicine, neonatology and paediatrics[1]. A purposive sampling strategy[2] was adopted in two NHS sites in the North of England. Fourteen interviews were conducted.

Accessing social care professionals was challenging. Challenges included barriers placed by some line managers who rejected being included in a study on TOPFA. A snowball sampling approach was found to be a more appropriate method of recruitment. Nine interviews were conducted. This research has used the umbrella term 'social care professionals' to define multiple different types of employment relating to those who work with people with impairments. This group included a range of roles including; disability care support workers, both mainstream and special needs teachers and workers involved with facilitating access into the community (both enabling independent living, and involvement in everyday activities). All participants had experience of working with people with impairments in a supportive social

---

[1] Additional information cannot be provided due to the sensitivity of the subject area and for confidentiality purposes.
[2] Participants selected and invited to participate due to meeting the inclusion criteria of the study.

For peer review only - http://bmjopen.bmj.com/site/about/guidelines.xhtml

context[1]. A mix of both male and female participants were recruited from both professional groups.

Interviews were transcribed verbatim. An inductive thematic analysis (19) was conducted on the data by LC. This approach allows the generation of themes to come from the data. Nvivo software was used for analysis. Analysis was conducted alongside data collection which allowed for the exploration of emergent themes. A random sample of interviews were coded separately by RG, to provide a qualitative equivalent of inter-rater reliability for the coding framework.

A favourable ethical opinion was received from the Newcastle and North Tyneside 2 Research Ethics Committee (10/H0907/50).

Results

Professionals' accounts suggested that they wanted to present an acceptable moral self-image, and that their discussions on TOPFA reflected this position. Most participants did not support unquestioningly the TOPFA for certain fetal anomalies, but depending on different ethical and moral arguments, they were able to overcome some objections.

For peer review only - http://bmjopen.bmj.com/site/about/guidelines.xhtml

Restoring normality: can it be fixed?

According to the medical model, the body is likened to a machine that can be fixed.(4) ‘Fixing’ in this context refers to ‘correcting’ or treating the anomaly to move toward what would be considered ‘normal’ or palliative. This aspect of biomedicine was important due to the assumptions participants made about automatic enrolment into medical intervention after a diagnosis of fetal anomaly. Subsequent interventions included further testing, to establish the extent of the anomaly, and medical treatment after birth. The data suggest that enrolment into medical intervention to ‘fix’ the anomaly is assumed and unquestioned in most instances.

Isolated cleft lip was a condition that participants, overall, deemed to be minor. This conclusion was based on the success rate of postnatal surgical intervention.

> *I personally think that, cleft lip is a fairly minor anomaly… that is treatable, and that has a good outcome (Medical Professional 20).*

> *I thought it was a joke how can anybody terminate a baby for having a cleft lip… especially the way medical science is now and you can get so much plastic surgery (Social Care Professional 1).*

For peer review only - http://bmjopen.bmj.com/site/about/guidelines.xhtml

Participants focused on the possibility of correcting the physical anomaly, concluding that cleft lip can be 'fixed' resulting in a normal life (and therefore an unacceptable or questionable justification for TOPFA). However, within both professional groups, some participants drew on other issues which could justify TOP, including maternal choice, and using Clause C of the HFEA if the pregnancy was under the 24-week threshold.

> *I think it should be a choice, and they should be given as much information as they possibly can… they should have that option (Social Care Professional 2).*

> *… if you can terminate a healthy baby just because the mother wants to, I don't see why you can't terminate a baby with a minor abnormality if the mother wants to. (Medical Professional 10).*

However, the ability to 'fix' an anomaly was coupled with other factors, which feature in the process of negotiating acceptability in this context. Down syndrome cannot be fixed, yet, for participants, this did not automatically equate to acceptable TOPFA. This may be linked to wider societal condemnation, but also coupled with the fact that Down syndrome was not conceptualised with suffering.

> *You don't suffer with Downs syndrome, Downs syndrome is only a problem to the people around you. (Medical Professional 12).*

For peer review only - http://bmjopen.bmj.com/site/about/guidelines.xhtml

A baby with Downs syndrome who didn't have any associated physical abnormalities, they didn't have cardiac, or heart or gut defect... I would perceive without any doubt that they're not gonna suffer at all. (Medical Professional 13).

For social care professionals, the concept of 'suffering' was also discussed in comparison to a 'normal' person with a difficult life even without impairment. Medical professionals were not overtly opposed to TOPFA for Down syndrome but were very concerned about ensuring parents knew the full implications of the anomaly. Many were, however, keen to distance themselves personally from the decision.

I would support parents that wanted to terminate a pregnancy for Down syndrome. I sort of have a view that they should be aware of you know, what Down syndrome is and… a lot of parents with Down babies are you know very grateful for having them. (Medical Professional 17).

Will there be pain?

Conceptualisations of pain were an important consideration for participants from both professional groups. Participants' threshold between acceptable and unacceptable levels of pain differed, depending on various factors, including the anomaly being

For peer review only - http://bmjopen.bmj.com/site/about/guidelines.xhtml

discussed, perceptions about length of life gained through the pain received, personal views and personal experiences. HLH was useful in teasing out these thresholds because of the need for surgical intervention for survival.(20) Pain will be a feature of a person affected by HLH, and pain was featured as a justification for TOPFA. The pain of ongoing surgery for HLH was conceptualised differently to the 'one off' surgery for isolated cleft lip for example. The notion of preventing a life filled with painful experiences was a key issue for participants:

> *If I was absolutely convinced there was an abnormality that was just gonna cause pain and distress and then death you know, at an incredibly young age, whatever that abnormality might be, then, they're the kind of cases that you'd be more convinced that you were absolutely doing the right thing. (Medical Professional 8).*

> *It's very difficult to find where the line is and I think probably... my own line... would be somebody who's in pain that can't be alleviated (Social Care Professional 14).*

HLH was deemed an acceptable reason for TOPFA by some social care professionals. This centred on the impact of the child, the medical interventions and the pain they would have to endure. Other social care professionals had different thresholds and

For peer review only - http://bmjopen.bmj.com/site/about/guidelines.xhtml

compared the treatment to people who have to have '*heart surgery all the time*' (Social Care Professional 4) and thus not an acceptable justification for TOPFA.

Overall, medical professionals were able to negotiate acceptable TOPFA for HLH. Thus, the HLH case study provided the basis for a more nuanced discussion of pain as a process – what level of pain was acceptable to put a child through, to get them to what was seen as a reasonable quality of life. The focus on a live birth with HLH is on surgical intervention but this was conceptualised as a permanent feature of an affected person's life as HLH cannot be 'fixed', only corrected in palliative terms. More surgical intervention will be required to sustain life for someone with HLH:

> *We're talking about long term, you're talking about palliation, so operations… that achieve a circulation but they do not fix the problem, a heart that operates on one pump, and eventually that will fail in some manner (Medical Professional 9).*

> *It's very likely that either the baby won't survive or will need lots of surgery which may have a high chance of not being successful (Medical Professional 11).*

The negotiating process exhibited by the participants seemed to regard certainty of significant pain as something that could straightforwardly justify acceptable TOPFA. A

For peer review only - http://bmjopen.bmj.com/site/about/guidelines.xhtml

normal life experience did not, for them, feature certainty of significant pain. There was also recognition of the necessity of medical intervention which may not be enough to guarantee long term survival. Therefore, both medical and social care professionals accepted HLH as a serious anomaly, with TOPFA conceptualised as a legitimate option for most participants. There were exceptions however, as two social care professionals (4 and 14) raised issues around placing a value on life. For them, it was immoral to deny a chance at life. Palliative care was also raised by two medical professionals as a legitimate option. For example, one professional stated:

> *I'd have a live born baby, take it home, cuddle it, you know, wait for it to die quietly... which is not the same thing as terminating it but also isn't the same thing as embarking on 35 years of, you know, horribly intensive, invasive medical involvement (Medical Professional 19).*

These participants' accounts suggested that they felt it was not necessarily in the best interests of the baby to intervene but that parents may also have moral objections to TOPFA. This option of palliative care '*can get you off both hooks*', (i.e. avoiding the decision to proceed with TOPFA whilst also preventing the baby from living a life of painful experiences). Palliative care was therefore seen as a route through the difficulties while maintaining an acceptable moral position and self-view.

For peer review only - http://bmjopen.bmj.com/site/about/guidelines.xhtml

Living a good life - Is it possible to be a 'normal' life trajectory?

The social construction of contemporary western society places high value on walking, and wheelchairs often symbolise impairment. The wheelchair cannot be hidden in the same way as, for example, bowel or bladder problems. The data show that a number of considerations influence participants' conceptualisations of the acceptability of TOPFA for spina bifida encompassing both visible and hidden elements; where the lesion is located, the presence of hydrocephalus, and mobility issues.

> *They realise that the child would need help with the bowel or walking, then, you know, may need a shunt and those things, then that is unacceptable… but when there's a lower defect, we give them the information (Medical Professional 5).*

Many medical and social care professionals had mixed opinions as to the acceptability of TOPFA for spina bifida. This variation stemmed from the dichotomy of spina bifida being a serious anomaly with serious consequences, and yet, with professionals speculating that if you asked a person affected by the condition if they would rather have not been born, the answer would likely be no.

For peer review only - http://bmjopen.bmj.com/site/about/guidelines.xhtml

It's very hard for me to stand there and look at someone with spina bifida who's, you know, wheelchair bound, and you know is kind of struggling with life, and say that their quality of life is poor (Medical Professional 8).

The data showed that many professionals in both groups indicated that they saw spina bifida as an acceptable reason for a TOPFA in some instances, or that they would not deny the parents the right to make that decision. Despite some personal misgivings as individuals, participants negotiated their way through the issues to avoid adopting a position that would deny choice to others;

… spina bifida, they are like serious physical conditions that that child's quality of life will not able to be the same as any other child, they're not gonna be able to fully enjoy aspects of life that other children do (Social Care Professional 23).

Many of our participants argued that any impairment would make life more difficult (to varying degrees), but that a positive life experience was still achievable. However, they also noted that the huge impact on the lives of family members should not be ignored. A diagnosis of fetal anomaly was seen as changing the life of the parents and siblings forever, thus affecting their 'normal' life trajectory. For example, the level of thought that needs to go into simple aspects of everyday life was discussed by social care professionals, and the inconvenience of unpredictability by medical professionals.

For peer review only - http://bmjopen.bmj.com/site/about/guidelines.xhtml

You can't just hop to the supermarket, and all nip down, you have to plan things around, is it gonna be a long walk, are we gonna be able to park the car closer, just simple things too, have we got pee bags, have we got pads, are they gonna need that (Social Care Professionals 2).

… that's what life is like, it's gonna be unpredictable, you're gonna be bringing them in on Christmas day or, you know, you'll plan a holiday and then your child will be ill, there's all sorts of things that happen (Medical Professional 20).

The absence of a "normal" life, however, did not always lead to negotiation of acceptable TOPFA. Down syndrome is not an impairment that can be hidden, unless the affected person is removed from society. In the UK, routine screening for Down syndrome is offered to all pregnant women.(21) The availability of a 'routine test' may in itself be a factor, reinforcing the view that TOPFA is a socially acceptable, widely available option. Like spina bifida, Down syndrome has been discussed as a serious anomaly with serious implications, however negotiating acceptable TOPFA with a moral justification proved difficult for some participants in both professional groups.

… obviously, it (Down syndrome) makes their life more difficult, but there are people that have difficult lives all the time, it doesn't mean they shouldn't have a life. (Social Care Professional 4).

For peer review only - http://bmjopen.bmj.com/site/about/guidelines.xhtml

You don't suffer with Down syndrome, Down syndrome is only a problem to the people around you (Medical Professional 12).

The positive experiences of those affected meant that some participants experienced difficulty in negotiating acceptable TOPFA for Down syndrome, and many concluded that Down syndrome was at least a questionable rationale for TOPFA. However, for those who were able to negotiate TOPFA as an acceptable outcome, the issue of societal condemnation was raised as a relevant factor, despite research suggesting that most women diagnosed with Down syndrome opt for TOPFA.(22) These professionals felt that the representations of Down syndrome in mainstream culture tended to reflect the positive experiences of those with Down syndrome and neglected the more negative experiences.

Discussion

This qualitative study found that both medical and social care professionals adopt classificatory practices which allow them to negotiate a view that TOPFA is an acceptable option, whilst maintaining a self-image they deem to be morally acceptable. These practices are not dissimilar despite the distinct professional groups, and their different levels of experience with: (a) decision making that leads to TOPFA, or (b) living with impairment. The similarities in their processes of negotiation may be

For peer review only - http://bmjopen.bmj.com/site/about/guidelines.xhtml

a result of them being shaped by commonly held social understandings of both impairment, TOP and TOPFA. Through discussions of fixing, pain and normal life expectations, professionals were able to negotiate instances of acceptable TOPFA while maintaining a self-image they deem acceptable morally. Thus, they navigate their way between the seriousness of the anomaly in question, perceived immorality of denying choices, and the felt unacceptability of TOPFA as a whole and for particular conditions. Those who indicated that TOP(FA) should still be an option raised several justifications for their position: (i) using Clause C if the pregnancy is under 24 weeks' gestation, therefore removing the anomaly as the primary justification for TOP; and (ii) placing heavier emphasis on maternal choice, by framing the denial of choice to women as being immoral. These reasons allowed professionals to either openly reject TOPFA for particular anomalies, or integrate additional moral arguments into the discussion that allowed them to accept TOP as a legitimate option.

This research has shown how professionals come to decisions about their views on acceptability in relation to TOPFA. The lack of a definition or consensus of terms such as 'substantial risk' and 'serious handicap' that determine whether TOPFA is legally permissible have been raised by others.(8, 23, 24) However, it is unlikely that a more focused definition would be welcome (23), as this would remove the ability to negotiate additional considerations as part of the decision making process. The lack of

For peer review only

For peer review only - http://bmjopen.bmj.com/site/about/guidelines.xhtml

categorical definitions does however, lead to interpretation and means decisions are open to subjective beliefs.(25) Given this decision is arguably based on a great number of complex factors, it is reasoned that it is not possible to have a 'one correct way' to assist parents in making this decision.(26) Our study findings have also shown that despite the presence of a serious anomaly, such circumstances do not automatically equate to a straightforward conceptualisation of TOPFA as acceptable. This is due to perceptions on suffering, pain and broader quality of life. Healthcare is often evaluated considering quality of life (27), yet there is no definition as to what this means. Those who are suffering from severe disease do not always report having a low quality of life (27), as indicated by both professional groups, in particular social care professionals. This suggests that individual experience and expectations are considered, alongside other aspects of the anomaly, in how professionals made sense of the concept of quality of life. Thus, the negotiation of acceptable TOPFA, necessarily factors in things outside of the individual anomaly itself and its biological impact. One example was the considerable impact on the whole family; acceptable TOPFA could be negotiated if the TOPFA was in the best interests of the family unit, which may include other children who may also suffer and miss out on a normal life experience because of the fetal anomaly. Thus, an understanding of what it means to live with impairment and disability is key in decision making processes, something medical professionals

For peer review only - http://bmjopen.bmj.com/site/about/guidelines.xhtml

involved in TOPFA are arguably less experienced with. For example, assumptions might be made about perceived burden (28), or that the presence of a disability automatically equates to being 'unhealthy'.(12)

Parents have also been found to have questions and concerns that are not addressed during counselling, in part due to the positioning of counselling within the medical model paradigm.(29) The inclusion of social care professionals in this study, will contribute to an increased understanding of differing perspectives. This is particularly significant given the similar views that have been presented across the two professional groups, which may suggest they reflect a perceived societal wide acceptance of TOPFA.

We propose that an acceptable TOPFA was determined by our participants regarding three key factors: whether a particular anomaly can be 'fixed' under the paradigm of biomedicine; what pain this 'fixing' will inflict; and whether there will be a 'normal' life trajectory with a sufficient degree of participation and fulfilment. The knowledge, meanings and interactions the different professional groups gain from their professional roles help shape their perspectives on TOPFA. The level of similarity may also be important in terms of assessing the extent to which knowledge about impairment, normality and suffering is constructed with reference to societal level factors.

For peer review only - http://bmjopen.bmj.com/site/about/guidelines.xhtml

This paper makes no claims to generalisability, especially given the findings have been collected in one geographical location. Social care professionals' views are underrepresented in this field of research and their inclusion has enabled their views to be represented. This research will add to ongoing discussions around TOPFA from the medical professional perspective. This is important given the first point of contact for many parents after a diagnosis of fetal anomaly is in a healthcare setting. While snowball sampling has issues regarding bias (30), it proved to be invaluable as a recruitment source in this research.

While medical professionals have been studied previously in relation to TOPFA, it is important to continue research to ensure that: (i) the evidence base remains up-to-date, especially in a continually changing society; and (ii) that the evidence available is used to inform effective guidelines that can work with existing clinical practices. Social care professionals are underrepresented in research relating to TOPFA, as well as social policy discussions, despite their knowledge and professional experience with people with impairments. This paper also offers perspectives of both these two professional groups, each associated with distinctive and different experiences. Despite these differences, the results showed their views to be remarkably similar when considering acceptable TOPFA. This may suggest a greater influence of societal wide views, which may need to be considered in any future research. it is also

For peer review only - http://bmjopen.bmj.com/site/about/guidelines.xhtml

important to note that no professional in the study suggested the legislation should be changed.

Acknowledgements

We would like to thank all the study participants for giving up their time to be interviewed.

Contribution statement

LC conceived and designed the research study, with RG, SR and JR. LC was responsible for the acquisition of data. LC coded and analysed all transcripts with RG coded a random sample. All authors were involved in the interpretation of the data. LC wrote the first draft of the manuscript and all authors were involved in subsequent revision. All authors approve the final manuscript.

Competing Interest statement

The authors declare that there is no conflict of interest.

Funding statement

This research was funded by a Medical Research Council/Economic and Social Research Council Integrated studentship, G0800128-3/1.

Data sharing statement

For peer review only - http://bmjopen.bmj.com/site/about/guidelines.xhtml

This research is a result of a PhD by LC. This PhD is available via the Newcastle University depository.

1. Human Fertilisation and Embryology Act 2008: Legislation.gov.uk; 2008 [cited 2014 25th January]. Available from: http://www.legislation.gov.uk/ukpga/2008/22/contents.
2. Department of Health. Abortion Statistics, England and Wales: 2016. 2017. Available from: https://www.gov.uk/government/uploads/system/uploads/attachment_data/file/618533/Abortion_stats_2016_commentary_with_tables.pdf.
3. Lafarge C, Mitchell K, Fox P. Women's experiences of coping with pregnancy termination for fetal abnormality. Qualitative Health Research. 2013;23(7):924-36.
4. Nettleton S. The sociology of health and illness. 3rd edition. Cambridge: Polity Press; 2013.
5. Persson M, Cnattingius S, Villamor E, Söderling J, Pasternak B, Stephansson O, et al. Risk of major congenital malformations in relation to maternal overweight and obesity severity: cohort study of 1.2 million singletons. BMJ. 2017;357.
6. Stothard KJ, Tennant PWG, Bell R, Rankin J. Maternal overweight and obesity and the risk of congenital anomalies: a systematic review and meta-analysis. JAMA. 2009;301(6):636-50.
7. Farsides B, Williams C, Alderson P. Aiming towards "moral equilibrium": health care professionals' views on working within the morally contested field of antenatal screening. Journal of Medical Ethics. 2004;30(5):505-9.
8. Fisher J, Lafarge C. Women's experience of care when undergoing termination of pregnancy for fetal anomaly in England. Journal of Reproductive and Infant Psychology. 2015;33(1):69-87.
9. Lotto R, Armstrong N, Smith LK. Care provision during termination of pregnancy following diagnosis of a severe congenital anomaly – A qualitative study of what is important to parents. Midwifery. 2016;43:14-20.
10. Statham H, Solomou W, Green J. Late termination of pregnancy: law, policy and decision making in four English fetal medicine units. BJOG: An International Journal of Obstetrics & Gynaecology. 2006;113(12):1402-11.
11. Garel M, Gosme-Seguret S, Kaminski M, Cuttini M. Ethical decision-making in prenatal diagnosis and termination of pregnancy: a qualitative survey among physicians and midwives. Prenatal Diagnosis. 2002;22(9):811-7.
12. Shakespeare T, Iezzoni LI, Groce NE. Disability and the training of health professionals. The Lancet. 2009;374(9704):1815-6.
13. Walker LV, Miller VJ, Dalton VK. The health-care experiences of families given the prenatal diagnosis of trisomy 18. Journal of Perinatology. 2008;28(1):12.

For peer review only

14. Guon J, Wilfond BS, Farlow B, Brazg T, Janvier A. Our children are not a diagnosis: the experience of parents who continue their pregnancy after a prenatal diagnosis of trisomy 13 or 18. American Journal of Medical Genetics Part A. 2014;164(2):308-18.
15. Marteau T, Nippert I, Hall S, Limbert C, Reid M, Bobrow M, et al. Outcomes of pregnancies diagnosed with Klinefelter syndrome: the possible influence of health professionals. Prenatal Diagnosis. 2002;22(7):562-6.
16. Marteau T, Drake H, Bobrow M. Counselling following diagnosis of a fetal abnormality: the differing approaches of obstetricians, clinical geneticists, and genetic nurses. Journal of Medical Genetics. 1994;31(11):864-7.
17. Curlin FA, Lawrence RE, Chin MH, Lantos JD. Religion, conscience, and controversial clinical practices. New England Journal of Medicine. 2007;356(6):593-600.
18. Crowe L. Termination of pregnancy for non-lethal fetal anomalies: Professional perspectives: Newcastle University; unpublihsed PhD thesis, 2014.
19. Braun V, Clarke V. Using thematic analysis in psychology. Qualitative Research in Psychology. 2006;3(2):77-101.
20. Satou GM, Klitzner TS. Hypoplastic left heart syndrome (HLHS). In: Yazdani S, McGhee S, R. S, editors. Chronic complex diseases of childhood: A practical guide for clinician. Boca Raton: Brown Walker Press; 2011. p. 24-8.
21. National Institute for Health and Clinical Excellence. Antenatal care: Routine care for the healthy pregnant women: National Institute for Health and Clinical Excellence.; 2008 [cited 2013 6th August]. Available from: http://publications.nice.org.uk/antenatal-care-cg62/aim.
22. Morris JK, Alberman E. Trends in Down's syndrome live births and antenatal diagnoses in England and Wales from 1989 to 2008: analysis of data from the National Down Syndrome Cytogenetic Register. BMJ. 2009;339:b3794.
23. Lotto R, Smith LK, Armstrong N. Clinicians' perspectives of parental decision-making following diagnosis of a severe congenital anomaly: a qualitative study. BMJ Open. 2017;7(5):e014716.
24. Savulescu J. Is current practice around late termination of pregnancy eugenic and discriminatory? Maternal interests and abortion. Journal of Medical Ethics. 2001;27(3):165-71.
25. Dommergues M, Mandelbrot L, Mahieu-Caputo D, Boudjema N, Durand-Zaleski I. Termination of pregnancy following prenatal diagnosis in France: how severe are the foetal anomalies? Prenatal Diagnosis. 2010;30(6):531-9.
26. McCoyd JLM. Critical Aspects of Decision-Making and Grieving After Diagnosis of Fetal Anomaly. In: Paley Galst J, Verp MS, editors. Prenatal and Preimplantation Diagnosis: The Burden of Choice. Cham: Springer International Publishing; 2015. p. 269-85.
27. Carr AJ, Gibson B, Robinson PG. Measuring quality of life: Is quality of life determined by expectations or experience? BMJ. 2001;322(7296):1240.

For peer review only - http://bmjopen.bmj.com/site/about/guidelines.xhtml

28. Parens E, Asch A. Disability rights critique of prenatal genetic testing: reflections and recommendations. Developmental Disabilities Research Reviews. 2003;9(1):40-7.
29. Hodgson J, Weil J. Talking about disability in prenatal genetic counseling: a report of two interactive workshops. Journal of Genetic Counseling. 2012;21(1):17-23.
30. Black JA, Champion DJ. Methods and issues in social research. Chichester: John Wiley and Sons; 1976.

For peer review only

Research Checklist.

1. Have you provided details of all of your co-authors? Is the information that you have entered into ScholarOne the same as the information on the manuscript title page?
   - Completed. Page 1.

2. Manuscript length and formatting: Have you checked that your manuscript doesn't exceed the requirements for word count, number of tables and/or figures, and number of references? Have you provided your abstract in the correct format? Have you supplied any required additional information for your article type, such as key messages.
   - The manuscript main body has a word count of 3999, which does not exceed the 4,000 limit. Page 1.

3. Tables: Have you embedded any tables into the main text? Have they been cited in the text? Have you provided appropriate table legends? Have you uploaded any lengthy tables as supplementary files for online publication?
   - Not applicable.

4. Figures: Have you uploaded any figures separately from the text? Have they been supplied in an acceptable format and are they of sufficient quality? Are they suitable for black and white reproduction (unless you intend to pay any required fees for colour printing)? Have the files been labelled appropriately? Have the figures been cited in the text? Have you provided appropriate figure legends?
   - Not applicable.

5. References: Have all of the references been cited in the text?
   - References have been uploaded using Endnote upon their use. Page 4-23.

6. Supplementary files and appendices: Have you supplied these in an acceptable format? Have they been cited in the main text?
   - Not applicable.

7. Statements: Have you included the necessary statements relating to contributorship, competing interests and funding, data sharing, patient consent and ethical approval?
   - Completed. Page 23.

8. Research reporting checklists: Have you either provided the appropriate statement for your study type, or explained why a checklist isn't required?
   - Not required for qualitative research.

9. Permissions: Have you obtained from the copyright holder to re-use any previously published material? Has the source been acknowledged?
   - Not applicable.

10. Reviewers: Have you provided the names of any preferred and non-preferred reviewers?
    - Completed. Submitted on the author submission forum.

For peer review only

BMJ Open

Negotiating acceptable termination of pregnancy for non-lethal fetal anomaly: A qualitative study of Professional perspectives.

Journal:	*BMJ Open*
Manuscript ID	bmjopen-2017-020815.R1
Article Type:	Research
Date Submitted by the Author:	22-Jan-2018
Complete List of Authors:	Crowe, Lisa; Newcastle University , Institute of Health and Society<br>Graham, Ruth; Newcastle University, School of Geography, Sociology and Politics<br>Robson, Stephen; Newcastle University, Institute of Cellular Medicine<br>Rankin, Judith; Newcastle University, Institute of Health & Society
**Primary Subject Heading**:	Reproductive medicine
Secondary Subject Heading:	Qualitative research
Keywords:	Fetal medicine < OBSTETRICS, Prenatal diagnosis < OBSTETRICS, Maternal medicine < OBSTETRICS, QUALITATIVE RESEARCH

SCHOLARONE™
Manuscripts

Negotiating acceptable termination of pregnancy for non-lethal fetal anomaly: A qualitative study of professional perspectives.

Lisa Crowe,[1] Ruth H Graham,[2] Stephen C Robson,[3] Judith Rankin.[1]

[1]Institute of Health & Society, Baddiley-Clark Building, Newcastle University, NE2 4AX.

[2] School of Geography, Politics and Sociology, 5th Floor Claremont Bridge Building, Newcastle University, NE1 7RU.

[3] Institute of Cellular Medicine, 3rd Floor William Leech Building, Newcastle University, NE2 4HH

Corresponding Author: Lisa Crowe. Lisa.Crowe@ncl.ac.uk

Word count (main body): 4428 .

For peer review only

For peer review only - http://bmjopen.bmj.com/site/about/guidelines.xhtml

Abstract

Objective: This study aims to explore the perspectives of professionals around the issue of termination of pregnancy for non-lethal fetal anomaly (TOPFA).

Methods: Semi-structured interviews were undertaken with medical professionals (14 consultants in fetal medicine, obstetrics, neonatology and paediatrics) and social care professionals (nine individuals with roles supporting people living with impairment) from the North East of England. Analysis adopted an inductive thematic approach facilitated by NVivo.

Results: The overarching theme to emerge from the interview data was of professionals, medical and social care, wanting to present an acceptable self-image of their views on TOPFA. Professionals' values on 'fixing', pain and 'normality' influenced what aspects of moral acceptability they gave priority to in terms of their standpoint and, in turn, their conceptualisations of acceptable TOPFA. Thus, if a termination could be defended morally, including negotiation of several key issues (including 'fixing', perceptions of pain and normality), then participants conceptualised TOPFA as an acceptable pregnancy outcome.

Conclusion: Despite different professional experiences, these professional groups were able to negotiate their way through difficult terrain to conceptualise TOPFA as a morally acceptable principle. While professionals have different moral thresholds, no one argued for a restriction of the current legislation. The data suggest that social care professionals also look at the wider social context of a person with an impairment when discussing their views regarding TOPFA. Medical professionals focus more on the individual impairment when discussing their views on TOPFA.

For peer review only - http://bmjopen.bmj.com/site/about/guidelines.xhtml

Keywords: UK; Termination of pregnancy; fetal anomaly; professionals; qualitative.

Strengths and Limitations:

- This qualitative study provides in-depth data on views on TOPFA from a previously unexplored professional group, social care professionals.
- The qualitative nature of this study allowed for the exploration of a sensitive research topic.
- The use of case studies provided tangible examples with which to explore key issues in the process of negotiating moral acceptability of TOPFA.
- This study was conducted in the North East of England so generalisation cannot be assumed but our conclusions are relevant and applicable in other contexts.

This work was supported by a Medical Research Council/ Economic and Social Research Council Integrated PhD studentship, grant number G0800128-3/1.

Introduction

Termination of pregnancy for fetal anomaly (TOPFA) is legal under the Abortion Act 1967, amended by the Human Fertilisation and Embryology Act (HFEA) (1) with no upper gestational limit if there "is a substantial risk that if the child were born it would suffer from such physical or mental abnormalities as to be seriously handicapped" (Clause E). Difficulties have been noted in defining terms such as "substantial" and "serious".(2-4) In 2016, 3,208 terminations were reportedly carried out under Clause E, 2% of the total number.(5) The

For peer review only - http://bmjopen.bmj.com/site/about/guidelines.xhtml

detection of fetal anomalies is likely to continue to increase due to improvements in fetal imaging and increasing risk factors for fetal anomalies (for example, obesity).(6-9) Fetal anomaly screening is offered to all pregnant women through the NHS Fetal Anomaly Screening Programme (FASP). The FASP defines a fetal anomaly as an abnormality which "may indicate the baby might die shortly after birth, conditions that may benefit from treatment before birth, to plan delivery in an appropriate hospital/Centre and/or to optimize treatment after the baby is born".(10) Complex issues emerge as medical professionals juggle multiple moral implications; judge the anomaly in question, offering choice, protecting themselves from prosecution while also providing care to parents.(11) These issues are compounded by the limited in-utero treatments available, thus reducing parental options to either TOPFA or continue with the affected pregnancy.(2) Thus, medical professionals working in prenatal diagnosis have the potential to influence decision making processes being made about an affected pregnancy; understanding professionals' views of TOPFA is therefore of crucial importance. This is especially significant given recent research that has found that care received by parents undergoing TOPFA was felt to not adequately meet their needs; for example, being caught between antenatal and postnatal care settings, yet belonging to neither.(12)

Personal views and experiences affect professional behaviour and the views of professionals impact on the pregnant woman. For example, research suggests that some women have felt they were counselled 'towards' TOPFA (13, 14), and other research has identified variations in counselling techniques.(15, 16) Religious affiliation has also been found to impact counselling practices.(17) If personal views impact on counselling practices, this may, in turn, influence decisions about TOPFA. Medical professionals providing TOPFA deal with complex information when deciding whether to offer TOPFA or not.(18, 19) However, their

For peer review only - http://bmjopen.bmj.com/site/about/guidelines.xhtml

knowledge and experience of living with disability and impairment tends to be more limited. This is argued to be of concern if assumptions about experience of impairment lead to disability being automatically equated to 'unhealthy'.(20) Similarly, those in the social care sector, involved in the support and care of those living with impairment, have more knowledge about experiences of impairment, but less insight into the decision making process that leads to TOPFA. Understanding the views of both medical and social care professionals about TOPFA is important not only to facilitate the provision of appropriate care and to provide support to those making reproductive decisions, but also to gain enhanced insight into how life with impairment is conceptualised from varied perspectives. Social care professionals have a limited voice in debates on TOPFA. Their views offer a different professional insight into what living with an impairment is like. To our knowledge, there is no research on the opinions of social care professionals on TOPFA.

This study aims to explore the perspectives of professionals around the issue of TOPFA.

Methods

This paper reports data collected as part of a larger study examining professionals' views on TOPFA.(21) This paper focuses on the qualitative data. Semi-structured interviews were used to collate data exploring professionals' perceptions of the complex issues surrounding TOPFA. Four fetal anomalies were selected as case studies; isolated cleft lip, hypoplastic left

For peer review only

heart (HLH), spina bifida and Down syndrome[1]. These examples ensured that discussions included reference to a range of conditions, affecting physical and intellectual capacity and with impact ranging from functionally minor to lethal[2].

Two groups were recruited to the study; medical and social care professionals. Medical professionals were consultants working in the fields of obstetrics, fetal medicine, neonatology and paediatrics[3]. A purposive sampling strategy[4] was adopted in two NHS sites in the North of England. Fourteen interviews were conducted.

Accessing social care professionals was challenging; a snowball sampling approach was found to be a more appropriate method of recruitment. Nine interviews were conducted. The umbrella term 'social care professionals' includes a range of roles; disability care support workers, both mainstream and special needs teachers and workers involved with facilitating access into the community (both enabling independent living, and involvement in everyday activities). All participants had experience of working with people with impairments in a supportive social context[3].

[1] Cleft lip arises when the upper lip fails to develop normally. As well as the pre-surgery disfigurement, infants may experience problems eating, speaking and hearing.(22). Surgery is available, but scarring is often evident and more extensive clefts need on-going input from dental and speech therapists. HLH occurs when the left side of the heart fails to develop. Without major heart surgery, HLH is fatal. Babies require multiple operations during childhood but only 65% survive to age 5 years.(23, 24) Spina bifida is a neural tube defect.(25) Medical intervention includes surgery to close the spina bifida and often to manage hydrocephalus, bowel and bladder interventions and, in many instances, devices to assist ambulation (e.g. braces) as well as psychosocial intervention. Downs syndrome is a chromosomal anomaly associated varying degrees of cognitive disability. (MacNair and Hicks, 2011). Improvements in management have resulted in an increase in the survival of affected individuals.(26-28). including those with other associated anomalies.(26)

[2] HLH is lethal without medical intervention.

[3] Additional information cannot be provided due to the sensitivity of the subject area and for confidentiality purposes.

[4] Participants selected and invited to participate due to meeting the inclusion criteria of the study.

For peer review only - http://bmjopen.bmj.com/site/about/guidelines.xhtml

Interviews were transcribed verbatim. An inductive thematic analysis (29) was conducted on the data by LC. This approach allows the generation of themes to come from the data. Nvivo software was adopted to support analysis. Analysis was conducted alongside data collection which allowed for the exploration of emergent themes. A random sample of interviews were coded separately by RG, to provide a qualitative equivalent of inter-rater reliability for the coding framework.

A favourable ethical opinion was received from the Newcastle and North Tyneside 2 Research Ethics Committee (10/H0907/50).

Results

The data revealed remarkably similar themes within both professional groups despite the very different occupational backgrounds. Thus, the data is presented by theme, rather than by professional group. Professionals' accounts suggested that they wanted to present an acceptable moral self-image, and that their discussions on TOPFA reflected this position. Most participants did not support unquestioningly the TOP for certain fetal anomalies, but depending on different ethical and moral arguments, they were able to overcome some objections.

Restoring normality: can it be fixed?

According to the medical model, the body is likened to a machine that can be fixed.(7) 'Fixing' in this context refers to 'correcting' or treating the anomaly to move toward what would be considered 'normal' or palliative. This aspect of biomedicine was important due to

For peer review only - http://bmjopen.bmj.com/site/about/guidelines.xhtml

For peer review only

the assumptions participants made about automatic enrolment into medical intervention after a diagnosis of fetal anomaly. Subsequent interventions included further testing, to establish the extent of the anomaly, and medical treatment after birth. The data suggest that enrolment into medical intervention to 'fix' the anomaly is assumed and unquestioned in most instances. The exception to this was Medical Professional 19 who discussed palliative care as a real option that is not well explored by medical professionals, however the reliance on expert knowledge was still revealed to be part of this option. Despite this, palliative care is still a treatment path, even if this is not to actively treat the impairment. Enrolment into medical intervention therefore is revealed by the data as seemingly the option, regardless of whether there is the possibility of a cure.

Isolated cleft lip was a condition that participants, overall, deemed to be minor. This conclusion was based on the success rate of postnatal surgical intervention.

> *I personally think that, cleft lip is a fairly minor anomaly… that is treatable, and that has a good outcome (Medical Professional 20).*

> *I thought it was a joke how can anybody terminate a baby for having a cleft lip… especially the way medical science is now and you can get so much plastic surgery (Social Care Professional 1).*

Participants focused on the possibility of correcting the physical anomaly, concluding that cleft lip can be 'fixed' resulting in a normal life (and therefore an unacceptable or questionable justification for TOPFA). However, within both professional groups, some participants drew on other issues which could justify TOP, including maternal choice, and using Clause C of the HFEA if the pregnancy was under the 24-week threshold.

For peer review only - http://bmjopen.bmj.com/site/about/guidelines.xhtml

I think it should be a choice, and they should be given as much information as they possibly can… they should have that option (Social Care Professional 2).

… if you can terminate a healthy baby just because the mother wants to, I don't see why you can't terminate a baby with a minor abnormality if the mother wants to. (Medical Professional 10).

However, the ability to 'fix' an anomaly was coupled with other factors, which feature in the process of negotiating acceptability in this context. Down syndrome cannot be fixed, yet, for participants, this did not automatically equate to acceptable TOPFA. This may be linked to wider societal condemnation, but also coupled with the fact that Down syndrome was not conceptualised with suffering.

You don't suffer with Downs syndrome, Downs syndrome is only a problem to the people around you. (Medical Professional 12).

A baby with Downs syndrome who didn't have any associated physical abnormalities, they didn't have cardiac, or heart or gut defect... I would perceive without any doubt that they're not gonna suffer at all. (Medical Professional 13).

For social care professionals, the concept of 'suffering' was also discussed in comparison to a 'normal' person with a difficult life even without impairment. Medical professionals were not overtly opposed to TOP for Down syndrome but were very concerned about ensuring parents knew the full implications of the anomaly. Many were, however, keen to distance themselves personally from the decision.

I would support parents that wanted to terminate a pregnancy for Down syndrome. I sort of have a view that they should be aware of you know, what Down syndrome is

For peer review only - http://bmjopen.bmj.com/site/about/guidelines.xhtml

and… a lot of parents with Down babies are you know very grateful for having them. (Medical Professional 17).

Will there be pain?

Conceptualisations of pain were an important consideration for participants from both professional groups. Participants' threshold between acceptable and unacceptable levels of pain differed, depending on various factors, including the anomaly being discussed, perceptions about length of life gained through the pain received, personal views and personal experiences. HLH was useful in teasing out these thresholds because of the need for surgical intervention for survival.(30) Pain will be a feature of a person affected by HLH, and pain was featured as a justification for TOPFA. The pain of ongoing surgery for HLH was conceptualised differently to the 'one off' surgery for isolated cleft lip for example. The notion of preventing a life filled with painful experiences was a key issue for participants:

If I was absolutely convinced there was an abnormality that was just gonna cause pain and distress and then death you know, at an incredibly young age, whatever that abnormality might be, then, they're the kind of cases that you'd be more convinced that you were absolutely doing the right thing. (Medical Professional 8).

It's very difficult to find where the line is and I think probably... my own line... would be somebody who's in pain that can't be alleviated (Social Care Professional 14).

HLH was deemed an acceptable reason for TOPFA by some social care professionals. This centred on the impact of the child, the medical interventions and the pain they would have to endure. Others however, compared treatment for HLH to people who have to have '*heart*

For peer review only - http://bmjopen.bmj.com/site/about/guidelines.xhtml

surgery all the time' (Social Care Professional 4) and thus not an acceptable justification for TOPFA.

Overall, medical professionals were able to negotiate acceptable TOP for HLH. Thus, the HLH case study provided the basis for a more nuanced discussion of pain as a process – what level of pain was acceptable to put a child through, to get them to what was seen as a reasonable quality of life. The focus on a live birth with HLH is on surgical intervention but this was conceptualised as a permanent feature of an affected person's life as HLH cannot be 'fixed', only corrected in palliative terms. More surgical intervention will be required to sustain life for someone with HLH:

> *We're talking about long term, you're talking about palliation, so operations… that achieve a circulation but they do not fix the problem, a heart that operates on one pump, and eventually that will fail in some manner (Medical Professional 9).*

> *It's very likely that either the baby won't survive or will need lots of surgery which may have a high chance of not being successful (Medical Professional 11).*

The negotiating process exhibited by the participants seemed to regard certainty of significant pain as something that could straightforwardly justify acceptable TOPFA. A normal life experience did not, for them, feature certainty of significant pain. There was also recognition of the necessity of medical intervention which may not be enough to guarantee long term survival. Therefore, both medical and social care professionals accepted HLH as a serious anomaly, with TOPFA conceptualised as a legitimate option for most participants. There were exceptions however, as two social care professionals (4 and 14) raised issues around placing a value on life. For them, it was immoral to deny a chance at life. Not actively

For peer review only

For peer review only - http://bmjopen.bmj.com/site/about/guidelines.xhtml

intervening was also raised by two medical professionals as a legitimate option. For example, one professional stated:

> *I'd have a live born baby, take it home, cuddle it, you know, wait for it to die quietly... which is not the same thing as terminating it but also isn't the same thing as embarking on 35 years of, you know, horribly intensive, invasive medical involvement (Medical Professional 19).*

These participants' accounts suggested that they felt it was not necessarily in the best interests of the baby to intervene but that parents may also have moral objections to TOPFA. This option of palliative care '*can get you off both hooks*', (i.e. avoiding the decision to proceed with TOPFA whilst also preventing the baby from living a life of painful experiences). Palliative care was therefore seen as a route through the difficulties while maintaining an acceptable moral position and self-view.

Is it possible to have a 'normal' life trajectory?

The social construction of contemporary western society places high value on walking, and wheelchairs often symbolise impairment. The wheelchair cannot be hidden in the same way as, for example, bowel or bladder problems. The data show that a number of considerations influence participants' conceptualisations of the acceptability of TOP for spina bifida encompassing both visible and hidden elements; where the lesion is located, the presence of hydrocephalus, and mobility issues.

For peer review only - http://bmjopen.bmj.com/site/about/guidelines.xhtml

> *They realise that the child would need help with the bowel or walking, then, you know, may need a shunt and those things, then that is unacceptable… but when there's a lower defect, we give them the information (Medical Professional 5).*

Many medical and social care professionals had mixed opinions as to the acceptability of TOP for spina bifida. This variation stemmed from the dichotomy of spina bifida being a serious anomaly with serious consequences, and yet, with professionals speculating that if you asked a person affected by the condition if they would rather have not been born, the answer would likely be no.

> *It's very hard for me to stand there and look at someone with spina bifida who's, you know, wheelchair bound, and you know is kind of struggling with life, and say that their quality of life is poor (Medical Professional 8).*

The data showed that many professionals in both groups indicated that they saw spina bifida as an acceptable reason for a TOPFA in some instances, or that they would not deny the parents the right to make that decision. Despite some personal misgivings as individuals, participants negotiated their way through the issues to avoid adopting a position that would deny choice to others;

> *… spina bifida, they are like serious physical conditions that that child's quality of life will not able to be the same as any other child, they're not gonna be able to fully enjoy aspects of life that other children do (Social Care Professional 23).*

Many of our participants argued that any impairment would make life more difficult (to varying degrees), but that a positive life experience was still achievable. However, they also noted that the huge impact on the lives of family members should not be ignored. A

For peer review only - http://bmjopen.bmj.com/site/about/guidelines.xhtml

diagnosis of fetal anomaly was seen as changing the life of the parents and siblings forever, thus affecting their 'normal' life trajectory. For example, the level of thought that needs to go into simple aspects of everyday life was discussed by social care professionals, and the inconvenience of unpredictability by medical professionals.

> *You can't just hop to the supermarket, and all nip down, you have to plan things around, is it gonna be a long walk, are we gonna be able to park the car closer, just simple things too, have we got pee bags, have we got pads, are they gonna need that (Social Care Professionals 2).*

> *… that's what life is like, it's gonna be unpredictable, you're gonna be bringing them in on Christmas day or, you know, you'll plan a holiday and then your child will be ill, there's all sorts of things that happen (Medical Professional 20).*

The absence of a "normal" life, however, did not always lead to negotiation of acceptable TOPFA. Down syndrome is not an impairment that can be hidden, unless the affected person is removed from society. In the UK, routine screening for Down syndrome is offered to all pregnant women.(31) The availability of a 'routine test' may in itself be a factor, reinforcing the view that TOPFA is a socially acceptable, widely available option. Like spina bifida, Down syndrome has been discussed as a serious anomaly with serious implications, however negotiating acceptable TOPFA with a moral justification proved difficult for some participants in both professional groups.

> *… obviously, it (Down syndrome) makes their life more difficult, but there are people that have difficult lives all the time, it doesn't mean they shouldn't have a life. (Social Care Professional 4).*

For peer review only - http://bmjopen.bmj.com/site/about/guidelines.xhtml

You don't suffer with Down syndrome, Down syndrome is only a problem to the people around you (Medical Professional 12).

The positive experiences of those affected meant that some participants experienced difficulty in negotiating acceptable TOP for Down syndrome, and many concluded that Down syndrome was at the least a questionable rationale for TOPFA. However, for those who were able to negotiate TOPFA as an acceptable outcome, the issue of societal condemnation was raised as a relevant factor, despite research suggesting that most women diagnosed with Down syndrome opt for TOPFA.(32) These professionals felt that the representations of Down syndrome in mainstream culture tended to reflect the positive experiences of those with Down syndrome and neglected the more negative experiences.

Discussion

This qualitative study found that both medical and social care professionals adopt classificatory practices which allow them to negotiate a view that TOPFA is an acceptable option, whilst maintaining a self-image they deem to be morally acceptable. These practices are not dissimilar despite the distinct professional groups, and their different levels of experience with: (a) decision making that leads to TOPFA, or (b) living with impairment. The similarities in their processes of negotiation may be a result of them being shaped by commonly held social understandings of both impairment, TOP and TOPFA. Through discussions of fixing, pain and normal life expectations, professionals were able to negotiate instances of acceptable TOPFA while maintaining a self-image they deem acceptable morally. Thus, they navigate their way between the perceived seriousness of the anomaly in question, perceived immorality of denying choices, and the felt unacceptability of TOPFA as

For peer review only - http://bmjopen.bmj.com/site/about/guidelines.xhtml

a whole and for particular conditions. Those who indicated that TOP(FA) should still be an option raised several justifications for their position: (i) using Clause C if the pregnancy is under 24 weeks' gestation, therefore removing the anomaly as the primary justification for TOP; and (ii) placing heavier emphasis on maternal choice, by framing the denial of choice to women as being immoral. These reasons enabled professionals to either openly reject TOP for particular anomalies, or integrate additional moral arguments into the discussion that allowed them to accept TOP as a legitimate option.

This research has shown how professionals come to decisions about their views on acceptability in relation to TOPFA. The lack of a definition or consensus of terms such as 'substantial risk' and 'serious handicap' that determine whether TOPFA is legally permissible have been raised by others.(2-4) However, it is unlikely that a more focused definition would be welcome (3), as this would remove the ability to negotiate additional considerations as part of the decision making process. The lack of categorical definitions does however, lead to interpretation and means decisions are open to subjective beliefs.(33) Given this decision is arguably based on a great number of complex factors, it is reasoned that it is not possible to have a 'one correct way' to assist parents in making this decision.(34) Our study findings have also shown that despite the presence of a serious anomaly, such circumstances do not automatically equate to a straightforward conceptualisation of TOPFA as acceptable. This is due to perceptions on suffering, pain and broader quality of life. Healthcare is often evaluated considering quality of life (35), yet there is no definition as to what this means. Those who are suffering from severe disease do not always report having a low quality of life (35), as indicated by both professional groups, in particular social care professionals. This suggests that individual experience and expectations are considered, alongside other aspects of the anomaly, in how professionals

For peer review only - http://bmjopen.bmj.com/site/about/guidelines.xhtml

made sense of the concept of quality of life. Thus, the negotiation of acceptable TOPFA, necessarily factors in things outside of the individual anomaly itself and its biological impact. One example was the considerable impact on the whole family; acceptable TOPFA could be negotiated if the TOPFA was in the best interests of the family unit, which may include other children who may also suffer and miss out on a normal life experience because of the fetal anomaly. Thus, an understanding of what it means to live with impairment and disability is key in decision making processes, something medical professionals involved in TOPFA are arguably less experienced with. For example, assumptions might be made about perceived burden (36), or that the presence of a disability automatically equates to being 'unhealthy'.(20)

Parents have also been found to have questions and concerns that are not addressed during counselling, in part due to the positioning of counselling within the medical model paradigm.(37) The inclusion of social care professionals in this study, will contribute to an increased understanding of how TOPFA is conceptualised because of the different contexts in which social care and medical professionals work. Comparing the views from individuals across the two professional groups is valuable because disabilities feature as a possible future in the work of one group, and a lived experience in the work of the other. These contrasting standpoints are important to note, given the similarity of the views described by individuals across the two professional groups. This similarity, despite quite different work experiences, may suggest that our participants interpret their differing work experiences with reference to a shared societal wide acceptance of TOPFA and women's choices.

We propose that, for our participants, the conceptualisation of an acceptable TOPFA decision was influenced by three key factors: whether a particular anomaly can be 'fixed'

For peer review only - http://bmjopen.bmj.com/site/about/guidelines.xhtml

under the paradigm of biomedicine; what pain this 'fixing' will involve, recognising that medical intervention can be painful; and whether it is reasonable to anticipate that the affected baby could have a 'normal' life trajectory – whether that be in terms of length of life, or life years with a meaningful degree of participation and fulfilment. Each of these elements played a part in the ways that participants explained their understandings of the extent to which TOPFA was a morally acceptable option. The knowledge, meanings and interactions the different professional groups gain from their professional roles help shape their perspectives on TOPFA. The level of similarity may also be important in terms of assessing the extent to which knowledge about impairment, normality and suffering is constructed with reference to societal level factors.

This paper makes no claims to generalisability, especially given the findings have been collected in one geographical location. This research will add to ongoing discussions around TOPFA from the medical professional perspective. This is important given the first point of contact for many parents after a diagnosis of fetal anomaly is in a healthcare setting. While snowball sampling has issues regarding bias (38), it proved to be invaluable as a recruitment source in this research.

The main contribution from our analysis stems from the comparison between accounts from two different professional groups, where the respective roles of disability in the working environments provides the possibility of a comparative analysis. However, in addition to this main aim, our analysis also contributes to the body of knowledge on each of the two professional groups. The existing knowledge on understandings of TOPFA in the two groups differs, so our work makes a slightly different contribution to each. While medical professionals have been studied previously in relation to TOPFA, it is important to continue

For peer review only - http://bmjopen.bmj.com/site/about/guidelines.xhtml

research to ensure that: (i) the evidence base remains up-to-date, especially in a continually changing society; and (ii) that the evidence available is used to inform effective guidelines that can work with existing clinical practices. Social care professionals are underrepresented in research relating to TOPFA, as well as social policy discussions, despite their knowledge and professional experience with people with impairments. This paper offers perspectives of both these two professional groups, each associated with distinctive and different experiences. Despite these occupational differences, the results showed their views to be remarkably similar when considering acceptable TOPFA. This may suggest a greater influence of societal wide views, which may need to be considered in any future research.

Acknowledgements

We would like to thank all the study participants for giving up their time to be interviewed.

Contribution statement

LC conceived and designed the research study, with RG, SR and JR. LC was responsible for the acquisition of data. LC coded and analysed all transcripts with RG coded a random sample. All authors were involved in the interpretation of the data. LC wrote the first draft of the manuscript and all authors were involved in subsequent revision. All authors approve the final manuscript.

Competing Interest statement

The authors declare that there is no conflict of interest.

Funding statement

This research was funded by a Medical Research Council/Economic and Social Research Council Integrated studentship, G0800128-3/1.

For peer review only - http://bmjopen.bmj.com/site/about/guidelines.xhtml

Data sharing statement

This research is a result of a PhD by LC. This PhD is available via the Newcastle University depository.

1. Human Fertilisation and Embryology Act. Human Fertilisation and Embryology Act 2008: Legislation.gov.uk; 2008 [Available from: http://www.legislation.gov.uk/ukpga/2008/22/contents.
2. Fisher J, Lafarge C. Women's experience of care when undergoing termination of pregnancy for fetal anomaly in England. Journal of Reproductive and Infant Psychology. 2015;33(1):69-87.
3. Lotto R, Smith LK, Armstrong N. Clinicians' perspectives of parental decision-making following diagnosis of a severe congenital anomaly: a qualitative study. BMJ open. 2017;7(5):e014716.
4. Savulescu J. Is current practice around late termination of pregnancy eugenic and discriminatory? Maternal interests and abortion. Journal of Medical Ethics. 2001;27(3):165-71.
5. Department of Health. Abortion Statistics, England and Wales: 2016. 2017 [Available from: https://www.gov.uk/government/uploads/system/uploads/attachment_data/file/618533/Abortion_stats_2016_commentary_with_tables.pdf.
6. Lafarge C, Mitchell K, Fox P. Women's experiences of coping with pregnancy termination for fetal abnormality. Qualitative Health Research. 2013;23(7):924-36.
7. Nettleton S. The sociology of health and illness. 3rd edition. Cambridge: Polity Press; 2013.
8. Persson M, Cnattingius S, Villamor E, Söderling J, Pasternak B, Stephansson O, et al. Risk of major congenital malformations in relation to maternal overweight and obesity severity: cohort study of 1.2 million singletons. BMJ. 2017;357.
9. Stothard KJ, Tennant PWG, Bell R, Rankin J. Maternal overweight and obesity and the risk of congenital anomalies: a systematic review and meta-analysis. Jama. 2009;301(6):636-50.
10. Fetal Anomaly Screening Programme. 2015 [Available from: https://www.gov.uk/government/uploads/system/uploads/attachment_data/file/421650/FASP_Standards_April_2015_final_2_.pdf.
11. Farsides B, Williams C, Alderson P. Aiming towards "moral equilibrium": health care professionals' views on working within the morally contested field of antenatal screening. Journal of Medical Ethics. 2004;30(5):505-9.
12. Lotto R, Armstrong N, Smith LK. Care provision during termination of pregnancy following diagnosis of a severe congenital anomaly – A qualitative study of what is important to parents. Midwifery. 2016;43:14-20.
13. Walker LV, Miller VJ, Dalton VK. The health-care experiences of families given the prenatal diagnosis of trisomy 18. Journal of Perinatology. 2008;28(1):12.
14. Guon J, Wilfond BS, Farlow B, Brazg T, Janvier A. Our children are not a diagnosis: the experience of parents who continue their pregnancy after a prenatal diagnosis of trisomy 13 or 18. American Journal of Medical Genetics Part A. 2014;164(2):308-18.
15. Marteau T, Nippert I, Hall S, Limbert C, Reid M, Bobrow M, et al. Outcomes of pregnancies diagnosed with Klinefelter syndrome: the possible influence of health professionals. Prenatal diagnosis. 2002;22(7):562-6.
16. Marteau T, Drake H, Bobrow M. Counselling following diagnosis of a fetal abnormality: the differing approaches of obstetricians, clinical geneticists, and genetic nurses. Journal of Medical Genetics. 1994;31(11):864-7.

For peer review only

For peer review only - http://bmjopen.bmj.com/site/about/guidelines.xhtml

17. Curlin FA, Lawrence RE, Chin MH, Lantos JD. Religion, conscience, and controversial clinical practices. New England Journal of Medicine. 2007;356(6):593-600.
18. Statham H, Solomou W, Green J. Late termination of pregnancy: law, policy and decision making in four English fetal medicine units. BJOG: An International Journal of Obstetrics & Gynaecology. 2006;113(12):1402-11.
19. Garel M, Gosme-Seguret S, Kaminski M, Cuttini M. Ethical decision-making in prenatal diagnosis and termination of pregnancy: a qualitative survey among physicians and midwives. Prenatal diagnosis. 2002;22(9):811-7.
20. Shakespeare T, Iezzoni LI, Groce NE. Disability and the training of health professionals. The Lancet. 2009;374(9704):1815-6.
21. Crowe L. Termination of pregnancy for non-lethal fetal anomalies: Professional perspectives: Newcastle University; 2014.
22. Mossey PA, Little J, Munger RG, Dixon MJ, Shaw WC. Cleft lip and palate. The Lancet. 2009;374(9703):1773-85.
23. Tennant PWG, Pearce MS, Bythell M, Rankin J. 20-year survival of children born with congenital anomalies: a population-based study. The lancet. 2010;375(9715):649-56.
24. Barron DJ, Kilby MD, Davies B, Wright JGC, Jones TJ, Brawn WJ. Hypoplastic left heart syndrome. The Lancet. 2009;374(9689):551-64.
25. Barf HA, Post MWM, Verhoef M, Jennekens-Schinkel A, Gooskens R, Prevo AJH. Restrictions in social participation of young adults with spina bifida. Disability and Rehabilitation. 2009;31(11):921-7.
26. Rankin J, Tennant PWG, Bythell M, Pearce MS. Predictors of survival in children born with Down syndrome: a registry-based study. Pediatrics. 2012:peds. 2011-3051.
27. Glasson EJ, Sullivan SG, Hussain R, Petterson BA, Montgomery PD, Bittles AH. The changing survival profile of people with Down's syndrome: implications for genetic counselling. Clinical genetics. 2002;62(5):390-3.
28. Leonard S, Bower CK, Petterson B, Leonard H. Survival of infants born with Down's syndrome: 1980-96. Paediatric and perinatal epidemiology. 2000;14(2):163-71.
29. Braun V, Clarke V. Using thematic analysis in psychology. Qualitative research in psychology. 2006;3(2):77-101.
30. Satou GM, Klitzner TS. Hypoplastic left heart syndrome (HLHS). In: Yazdani S, McGhee S, R. S, editors. Chronic complex diseases of childhood: A practical guide for clinician. Boca Raton: Brown Walker Press; 2011. p. 24-8.
31. National Institute for Health and Clinical Excellence. Antenatal care: Routine care for the healthy pregnant women: National Institute for Health and Clinical Excellence.; 2008 [Available from: http://publications.nice.org.uk/antenatal-care-cg62/aim.
32. Morris JK, Alberman E. Trends in Down’s syndrome live births and antenatal diagnoses in England and Wales from 1989 to 2008: analysis of data from the National Down Syndrome Cytogenetic Register. Bmj. 2009;339:b3794.
33. Dommergues M, Mandelbrot L, Mahieu-Caputo D, Boudjema N, Durand-Zaleski I. Termination of pregnancy following prenatal diagnosis in France: how severe are the foetal anomalies? Prenatal diagnosis. 2010;30(6):531-9.
34. McCoyd JLM. Critical Aspects of Decision-Making and Grieving After Diagnosis of Fetal Anomaly. In: Paley Galst J, Verp MS, editors. Prenatal and Preimplantation Diagnosis: The Burden of Choice. Cham: Springer International Publishing; 2015. p. 269-85.
35. Carr AJ, Gibson B, Robinson PG. Measuring quality of life: Is quality of life determined by expectations or experience? BMJ: British Medical Journal. 2001;322(7296):1240.
36. Parens E, Asch A. Disability rights critique of prenatal genetic testing: reflections and recommendations. Developmental Disabilities Research Reviews. 2003;9(1):40-7.
37. Hodgson J, Weil J. Talking about disability in prenatal genetic counseling: a report of two interactive workshops. Journal of genetic counseling. 2012;21(1):17-23.

For peer review only - http://bmjopen.bmj.com/site/about/guidelines.xhtml

38. Black JA, Champion DJ. Methods and issues in social research. Chichester: John Wiley and Sons; 1976.

For peer review only

For peer review only - http://bmjopen.bmj.com/site/about/guidelines.xhtml

Research Checklist.

1. Have you provided details of all of your co-authors? Is the information that you have entered into ScholarOne the same as the information on the manuscript title page?
   - Completed. Page 1.

2. Manuscript length and formatting: Have you checked that your manuscript doesn't exceed the requirements for word count, number of tables and/or figures, and number of references? Have you provided your abstract in the correct format? Have you supplied any required additional information for your article type, such as key messages.
   - The manuscript main body has a word count of 3999, which does not exceed the 4,000 limit. Page 1.

3. Tables: Have you embedded any tables into the main text? Have they been cited in the text? Have you provided appropriate table legends? Have you uploaded any lengthy tables as supplementary files for online publication?
   - Not applicable.

4. Figures: Have you uploaded any figures separately from the text? Have they been supplied in an acceptable format and are they of sufficient quality? Are they suitable for black and white reproduction (unless you intend to pay any required fees for colour printing)? Have the files been labelled appropriately? Have the figures been cited in the text? Have you provided appropriate figure legends?
   - Not applicable.

5. References: Have all of the references been cited in the text?
   - References have been uploaded using Endnote upon their use. Page 4-23.

6. Supplementary files and appendices: Have you supplied these in an acceptable format? Have they been cited in the main text?
   - Not applicable.

7. Statements: Have you included the necessary statements relating to contributorship, competing interests and funding, data sharing, patient consent and ethical approval?
   - Completed. Page 23.

8. Research reporting checklists: Have you either provided the appropriate statement for your study type, or explained why a checklist isn't required?
   - Not required for qualitative research.

9. Permissions: Have you obtained from the copyright holder to re-use any previously published material? Has the source been acknowledged?
   - Not applicable.

10. Reviewers: Have you provided the names of any preferred and non-preferred reviewers?
    - Completed. Submitted on the author submission forum.

For peer review only
